# Impact of Relaxing Covid-19 Social Distancing Measures on Rural North Wales: A Simulation Analysis

**DOI:** 10.3389/fpubh.2020.562473

**Published:** 2020-12-14

**Authors:** Rhodri P. Hughes, Dyfrig A. Hughes

**Affiliations:** ^1^Glan Clwyd High School, Saint Asaph, United Kingdom; ^2^Centre for Health Economics and Medicines Evaluation, Bangor University, Bangor, United Kingdom

**Keywords:** Covid-19, rural health, health policy, public health, infectious disease

## Abstract

**Background:** Social distancing policies aimed to limit Covid-19 across the UK were gradually relaxed between May and August 2020, as peak incidences passed. Population density is an important driver of national incidence rates; however peak incidences in rural regions may lag national figures by several weeks. We aimed to forecast the timing of peak Covid-19 mortality rate in rural North Wales.

**Methods:** Covid-19 related mortality data up to 7/5/2020 were obtained from Public Health Wales and the UK Government. Sigmoidal growth functions were fitted by non-linear least squares and model averaging used to extrapolate mortality to 24/8/2020. The dates of peak mortality incidences for North Wales, Wales and the UK; and the percentage of predicted mortality at 24/8/2020 were calculated.

**Results:** The peak daily death rates in Wales and the UK were estimated to have occurred on the 14/04/2020 and 15/04/2020, respectively. For North Wales, this occurred on the 07/05/2020, corresponding to the date of analysis. The number of deaths reported in North Wales on 07/05/2020 represents 33% of the number predicted to occur by 24/08/2020, compared with 74 and 62% for Wales and the UK, respectively.

**Conclusion:** Policies governing the movement of people in the gradual release from lockdown are likely to impact significantly on areas–principally rural in nature–where cases of Covid-19, deaths and immunity are likely to be much lower than in populated areas. This is particularly difficult to manage across jurisdictions, such as between England and Wales, and in popular holiday destinations.

## Introduction

The Severe Acute Respiratory Syndrome Coronavirus-2 (SARS-CoV-2) has resulted in 20.7 m cases of Covid-19 worldwide (as of 14th August 2020) (1). Declared a pandemic by the World Health Organization on February the 11th 2020, measures to contain the spread of SARS-CoV-2 has seen most countries impose social distancing measures including restrictions on travel, work and closure of non-essential services. On the 23rd of March a lockdown was introduced in the United Kingdom (UK) to limit further spread of the virus.

Lockdown measures were aimed to suppress viral transmission, maintain a functioning health service, and reduce mortality. The UK Prime Minister, Boris Johnson, announced some easing of the lockdown measures for England on the 10th May 2020. With devolved powers to enforce measures to control movement of people in response to Covid-19, the governments of Wales and Scotland retained their social distancing measures until 1st June 2020. Differences in policies between countries within the UK reflect geographical differences in disease incidence, prevalence and the reproduction number, *R*_*t*_, which was estimated on the 10th May 2020 to be between 0.5 and 0.9 across the UK, but nearer to 1 in Scotland, and 0.8 in Wales (2).

During the initial phase of the first wave of Covid-19 cases across the UK (March to June 2020), social distancing policies applied at national levels, and did not reflect local variations in detected cases. Within Wales, for instance, the incidence of Covid-19 on 10th May 2020 varied substantially, with 446 cases per 100,000 in the South East (more populated, urban areas) to 247 cases per 100,000 in the North (sparsely populated and more rural) (3). Policies driven largely by changes in transmission rates in populated areas (which had mainly peaked by early May), might not have been applicable to rural areas (where cases had not yet peaked). Consequently, transmission caused by movements of people within and between UK countries may have been mitigated had local contexts been considered sooner. The introduction of local measures did not occur until 29th June 2020, in response to a spike in the number of cases in Leicester, England.

As a case in point, North Wales is primarily a rural region, with the north-west, in particular, being sparsely populated (<50 people /km^2^), and reliant on the tourism and agricultural economies. North Wales is a popular holiday destination, especially to visitors from the neighboring Liverpool-Manchester megalopolis (population 5.6 million). Over 3.9 million people visited the Snowdonia National Park alone in 2015 (4); and there are more than 5,000 s homes in north-west Wales, where 1 in 3 properties are sold to residents from outside the region. North Wales is served by a unitary health authority (Betsi Cadwaladr University Health Board, BCUHB), providing primary, secondary, community, and social care to 696,300 inhabitants. Increases in the population numbers risk placing pressure on the 3 district general hospitals that have 31 intensive care beds. In response to Covid-19, however, an additional 930 bed spaces have been made available via regional temporary hospitals.

During the weekend prior to the lockdown (21st−22nd March 2020) record numbers of tourists were reported to visit Snowdonia. The Snowdonia National Park Authority described an “unprecedented scene” which saw hundreds of people walking up Wales' highest mountain in what the authority said was “the busiest visitor day in living memory” (5). During this period there was also a surge in the number of people relocating—mainly from the north-west of England—to their second homes in North Wales. A few days immediately following the easing of the lockdown in England (13th May 2020), there were reports of holiday parks being “flooded” with booking requests, despite more strict laws applying in Wales (6).

The aim of the present analysis was to assess whether the trajectory of Covid-19 related mortality rates reported in BCUHB up to the date of easing of the lockdown in England mirror those for Wales, and UK as a whole. A comparison of forecasted and observed mortality to the end of the first wave (24th August 2020) provided a basis to assess differences in the rate of increase of deaths, timing of peak rates, and decline that may indicate whether earlier implementation of local policies would have been appropriate.

## Methods

### Data

Mortality figures for people with a positive test for Covid-19 were obtained from Public Health Wales (3) and the UK Government (7). Both datasets include patients who may have died from other causes, and exclude the deaths of people who were not tested, or who might have died from (or with) Covid-19 but did not tested positive.

Data for the UK and Wales were obtained from the 08/03/2020 and 18/03/2020, respectively, to the 07/05/2020. Data for BCUHB were obtained between the 20/03/2020 and the 07/05/2020; however, daily data for BHUHB were missing between 21/03/2020 to the 23/04/2020 because of a data reporting error and the Health Board reported all of the deaths between these dates on the 24/04/2020. Prior to 21/03/2020, there were fewer than 5 cases of deaths, this being the threshold for disclosing information to avoid de-anonymization.

### Analysis

Missing daily data for BCUHB were imputed using the predictions from an exponential function fitted to observed data points. This expression, $Deaths\left(t_{imp}\right)=1.7233^{*}exp^{0.0819^{*}t}$ was assumed to be applicable for historic data during the exponential growth phase of transmission. Cumulative mortality to 7th May 2020 was modeled using a range of sigmoidal growth functions: logistic, S-Shape, Richards, Weibull, and Gompertz functions, which are defined below:

(1) $$\begin{array}{l} Logistic\;Deaths\left(t_{L}\right)=\frac{a}{1+b^{*}exp^{-c*t\;}} \end{array}$$

(2) $$\begin{array}{l} S-Shape\;Deaths\left(t_{S}\right)=exp^{a+\left(\frac{b}{t}\right)} \end{array}$$

(3) $$\begin{array}{l} Weibull\;Deaths\left(t_{W}\right)=a-b*exp^{-c*t^{d}} \end{array}$$

(4) $$\begin{array}{l} Gompertz\;Deaths\left(t_{G}\right)=\;a*exp^{-exp^{b-c*t}} \end{array}$$

(5) $$\begin{array}{l} Richards\;Deaths\left(t_{R}\right)=\frac{a}{{\left(1+exp^{b-c*t}\right)}^{\frac{1}{d}}} \end{array}$$

Each were fitted to the data by least squares using the non-linear regression function (CurveFit) in Stata version 13 (StataCorp, College Station, TX) (8) to estimate parameters a, b, c, d for each equation. Modeling uncertainty was considered using unweighted model averaging.

The date of peak rate of deaths, corresponding to the steepest incline in the rate of cumulative deaths, was derived from the model averaging forecast. The modeled cumulative number of deaths by 24th August 2020 for each region (BCUHB, Wales, United Kingdom) was recorded, and the number of deaths to 07/05/2020 was expressed as a percentage of these values. Comparisons were made with observations available up to 24th August 2020.

## Results

**Figure 1:**
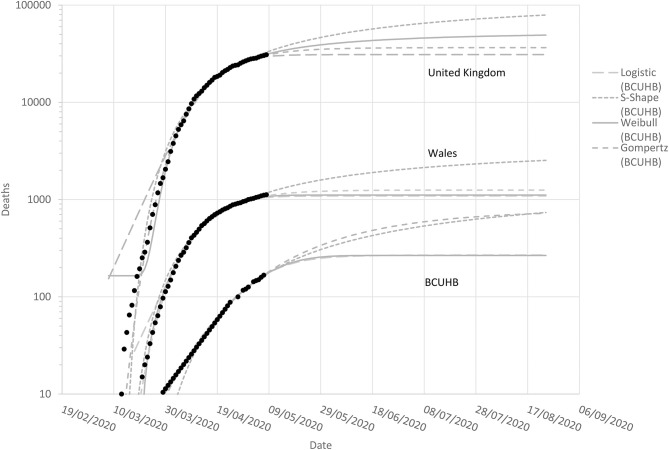
Observed (to 7th May 2020) and modeled cumulative mortality in Covid-19 positive patients in the UK **(top)**, Wales **(middle)**, and BCUHB **(bottom)**.

**Figure 2:**
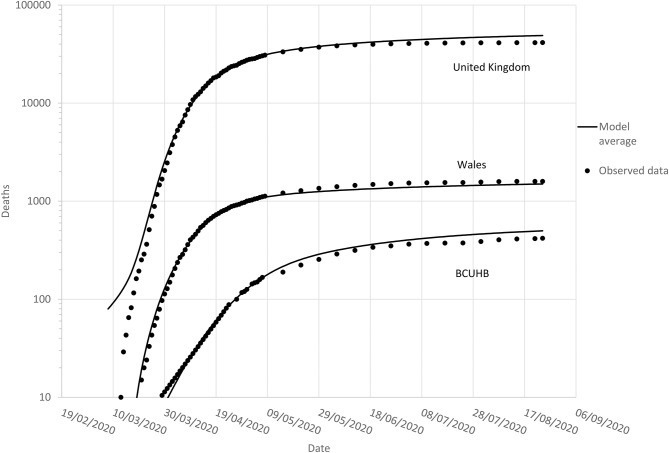
Observed (to 24th August 2020) and model-averaged forecasted cumulative mortality in Covid-19 positive patients in the UK **(top)**, Wales **(middle)**, and BUCHB **(bottom)**.

Convergence in the non-linear curve fitting was achieved for all functions. However, the parameter estimates for the Richards model indicated equivalence to the Gompertz model. This occurs under certain conditions when parameter *d* in equation 5 approaches zero, given that the Gompertz model is a special case of Richards model. For this reason, simulations involving the Richards model were not undertaken. The model parameter estimates and associated standard errors are presented in Table 1. Figure 1 depicts the cumulative growth in mortality, with each of the four models superimposed on the observed data used for model fitting. Figure 2 presents the modeled average nowcast (to 7th May 2020) with reported daily cases of mortality; and forecasted figures with weekly observed data to 24th August 2020.

**Table 1:** Parameter estimates for each model.

	**Model parameters**				
**Model**	**a**	**b**	**c**	**d**	***r^**2**^***
**BCUHB**					
Logistic	268.37 (16.38)	214.94 (8.22)	0.10 (23.46)		1.000
S-curve	7.44 (74.69)	−142.46 (−25.55)			0.999
Weibull	266.22 (6.96)	260.72 (6.66)	0.00 (1.05)	4.00 (14.12)	0.999
Gompertz	779.23 (3.63)	2.12 (38.98)	0.03 (7.58)		0.999
Richards	261.51 (4.44)	5.67 (2.20)	0.10 (2.48)	1.07 (1.78)	1.000
**Wales**					
Logistic	1,091.76 (87.69)	172.05 (9.05)	0.14 (39.03)		0.999
S-Curve	8.28 (281.45)	−75.39 (−53.85)			0.998
Weibull	1,112.74 (93.47)	1,141.45 (71.94)	0.00 (3.97)	3.18 (45.04)	0.999
Gompertz	1,252.46 (166.53)	2.56 (122.42)	0.07 (95.01)		1.000
Richards	1,252.33 (166.62)	−5.20 (n/a)	0.07 (95.08)	0.00 (47.81)	1.000
**UK**					
Logistic	30,984.59 (77.54)	229.88 (8.55)	0.14 (38.32)		0.998
S-curve	11.78 (425.55)	−86.02 (−63.81)			0.998
Weibull	164.84 (3.77)	−53,188 (−48.48)	1,130.74 (8.54)	−1.85 (−51.00)	1.000
Gompertz	36,523 (132.65)	2.65 (112.48)	0.07 (84.31)		1.000
Richards	36,521 (132.66)	−5.85 (n/a)	0.07 (84.33)	0.00 (42.46)	1.000

*Data in parentheses are the standard errors*.

The peak daily death rate in Wales was modeled to have occurred on the 14/04/2020 (range 11/04/2020–15/04/2020). Peak daily deaths for the UK occurred on 15/04/2020 (range 12/04/2020–20/04/2020)—both indicating that the first peaks for daily deaths had passed by the easing of the lockdowns in each country. For BCUHB, the peak for daily deaths was modeled to have occurred on the 07/05/2020 (range 02/05/2020–26/05/2020), corresponding to the date for which data were available at the time of analysis. This meant that the date of peak daily deaths for BCUHB was highly uncertain at the time decisions were made to relax the lockdown restrictions.

As of 07/05/2020, the number of deaths reported for BCUHB (167) represented 33% (range 23–63%) of the total forecasted cumulative number for 24th August 2020, suggesting that the region was not yet halfway in terms of absolute numbers of deaths in Covid-19 positive patients. By contrast, deaths across Wales was predicted to be 74% (range 44–100%) of the total, and the UK 62% (range 38–98%).

Based on data up to 7th May 2020, the total forecasted number deaths for the UK, Wales, and BCUHB by 24th August 2020 were, respectively, 49,107 (range 30,985–79,009), 1,497 (1,092–2,530), and 499 (266–736). The recorded numbers of deaths by this date were 41,443, 1,594, and 418, respectively.

## Discussion

The analysis demonstrated that parsimonious models of sigmoidal growth provided good fits to observed data up to 7th May 2020 on Covid-19 mortality across the UK, Wales, and North Wales. Averaging these models addressed key modeling uncertainties; and allowed forecasting that provided a reasonable measure of the scale of the first wave of the Covid-19 outbreak up to the 24th August 2020.

Modeling of data up to the 7th May 2020 suggested that the rate of Covid-19 positive deaths in Wales and the UK had already peaked, although there was predicted significant mortality in the weeks and months that followed over the course of the first wave, consistent with multiple other forecast models of Covid-19 (9). The situation was found to be different in North Wales, however, where there remained significant uncertainty concerning the timing of peak mortality. During this time (May 2020), concerns that the incidence of new cases may be rising at a higher rate than the remainder of Wales, coupled with the ≥2 week lag in mortality, implied that reducing strict controls on population movement may have been detrimental to the region's population health.

The fragility of rural North Wales in dealing with Covid-19 in the context of substantial increases in holidaymakers and second home residents is significant. The May 10th announcement of the relaxation in the lockdown for England, included freedom for exercise and outdoor activity, “irrespective of distance.” While Wales was still in lockdown during this period, the Welsh Government ruled that stopping people breaking Welsh coronavirus lockdown laws was not a “real option.” As it transpired, a relaxation of the lockdown in Wales followed a few weeks later (1st June 2020), but even by then, the peak in mortality had only just passed in North Wales.

Other factors might also contribute to differential rates of transmission and mortality. An important consideration is population demographics. Between 1997 and 2017, the proportion of the population aged 65 and over in North Wales increased from 19 to 23%, which is significantly higher than the UK average of 18% in 2018. This will have no doubt contributed to increased—if not delayed—death rates in North Wales.

Our analysis has strengths in consideration of multiple sigmoidal growth functions, contrasting with many others, including the influential Institute for Health Metrics and Evaluation (IHME) modeling which relies on a single model, namely the ERF error function. Their approach has been criticized as predictions are extremely labile since new data are included on a daily basis (10). Neither our model nor the IHME model is a disease transmission model, and this represents a limitation. Although in predicting mortality (as opposed to cases), SEIR compartmental models (representing susceptible, exposed, infectious, recovered) may be less reliable. The Covid-19 mortality forecasts made by the US Centers for Disease Control and Prevention are based on an “ensemble” forecast which combines independently developed forecasts into one aggregate forecast to improve prediction (11). This is equivalent to our model averaging approach, although it may be preferable to weight models based on historical performance (12). Model averaging benefits from possible reduction of predictive error. However, the confidence bounds for averaged models are not readily calculable, hence our presentation of the range of outputs from each individual model as a conservative estimate. A further limitation relates to the data, as not all Covid-19 deaths are reported in NHS and Government figures. Estimations of excess mortality are a more robust estimate of the overall impact of Covid-19, as these are inclusive also of wider impacts of hospital pressures and cancellation of elective procedures.

In conclusion, there were differences in the rates of Covid-19 related mortality across regions of the UK during the first wave in 2020. This may indicate that local measures could be more suited to target spikes in disease incidence. It also suggests that policies governing the movement of people following periods of lockdown might impact differentially depending on such factors as population density and demographics.

## Data Availability Statement

The original contributions presented in the study are included in the article/supplementary materials, further inquiries can be directed to the corresponding author/s.

## Ethics Statement

Ethical review and approval was not required for the study on human participants in accordance with the local legislation and institutional requirements. Written informed consent from the participants' legal guardian/next of kin was not required to participate in this study in accordance with the national legislation and the institutional requirements.

## Author Contributions

RH and DH made substantial contributions to the conception and design, acquisition of data, and analysis and interpretation of data. RH drafted the article and gave final approval of the version to be published. DH revised the article critically for important intellectual content and gave final approval of the version to be published.

## Conflict of Interest

The authors declare that the research was conducted in the absence of any commercial or financial relationships that could be construed as a potential conflict of interest.
